# *Editorial Commentary*: Fifteen Years of Protection by Meningococcal C Conjugate Vaccines: Lessons From Disease Surveillance

**DOI:** 10.1093/cid/ciu599

**Published:** 2014-07-28

**Authors:** Martin C. J. Maiden, Jenny M. MacLennan

**Affiliations:** Department of Zoology, University of Oxford, United Kingdom

**Keywords:** *Neisseria meningitidis*, vaccination, herd immunity, carriage, epidemics

**(See the Major Articles by Sadarangani et al on pages 1208–15, and Bijlsma et al on pages 1216–21.)**

Despite the extensive advances that have been made in biomedical sciences in the past few decades, the development and implementation of novel vaccines remains a highly pragmatic and uncertain endeavor. Although the concept of vaccination is >200 years old and was formalized by Pasteur >100 years ago, progress in the development of vaccines remains comparatively slow and has not accelerated in the way seen in virtually all other areas of technology. The many reasons for this include safety concerns arising from the administration of vaccinations to otherwise healthy individuals, often infants or children, and our inability to predict reliably the behavior of a novel human vaccine at the population level from data gathered in laboratory experiments or even during phase I or phase II trials of human subjects [1]. Although large placebo-controlled double-blind phase III trials can provide useful information in both regards, these are very expensive, prohibitively so if the disease is rare, and even very large studies may be insufficiently powered to detect population effects [1].

It is often the case, therefore, that vaccines are introduced without full knowledge of their likely impact, particularly without knowledge of how population effects might be best exploited in vaccination schedules. Consequently, it is important to ensure that enhanced disease surveillance is in place before, during, and after any vaccine introduction to evaluate vaccine impact post hoc and enable immunization schedules to be modified as necessary. Two articles in this issue [2, 3], describing the effectiveness of meningococcal serogroup C conjugate (MCC) vaccines during a 15-year period in the Netherlands and Canada, demonstrate the lasting value of such surveillance data in understanding how a vaccine works. They further illustrate how such information can be used to refine and improve implementation, even years after vaccine introduction. For MCC vaccines this was especially important, because no phase III efficacy trials had been conducted before implementation.

Compared with many of the current challenges in vaccinology [1], vaccines against the serogroup C meningococcus presented a relatively straightforward problem [4]. The expression of a capsular polysaccharide is strongly associated with the invasive phenotype in meningococci, with only a subset of the 12 known capsular variants (those corresponding to serogroups A, B, C, W, X, and Y) responsible for most epidemic and endemic disease [5]. Good antibody responses against capsular antigens were demonstrated to be important in protection against meningococcal disease in the 1960s [6], and the development of protein-polysaccharide conjugation technology in the 1980s [7] resulted in very safe, well-tolerated vaccines that were capable of eliciting strong anamnestic antibody responses. The successful implementation of the *Haemophilus influenzae* type b vaccines in the early 1990s provided evidence of the utility of such vaccines [8], paving the way for the development and implementation of highly effective meningococcal vaccines [9].

In most high-income countries, and many low- and middle-income countries outside the African meningitis belt, meningococcal disease is rare, with periods of low incidence punctuated by increased incidence, which may include localized disease outbreaks or hyperendemics [5]. For reasons that remain unclear, during the 1990s there were sharp increases in the number of cases of serogroup C disease caused by meningococci belonging to the hyperinvasive sequence type 11 complex in a number of countries. These increases were especially troubling because they were characterized by high attack rates in young adults and were frequently associated with high mortality and disease outbreaks in educational settings, such as schools and universities [10]. This increase in disease incidence stimulated the accelerated introduction of the MCC vaccines, which had just been developed, with the United Kingdom National Health Service implementing the first national immunization program in 1999 [11]. This was followed by introductions in other countries, including the Netherlands and Canada [12].

Many of the countries that implemented the MCC vaccines, including the United Kingdom, the Netherlands, and Canada, had means of meningococcal disease surveillance in place before and after implementation, at least to the level of serogroup, enabling the impact of the MCC vaccines to be addressed [13–15]. In the United Kingdom, a large-scale carriage study was also undertaken during vaccine introduction, allowing direct measurement of vaccine impact on the asymptomatic carriage of meningococci [16] and assessment of whether serogroup replacement occurred [17]; fortunately, for reasons that remain unclear, replacement that would have compromised the vaccine's effect did not occur [18]. Additional opportunities to learn about these vaccines after implementation were provided by the fact that these countries adopted different immunization strategies. In Canada, different provinces introduced the MCC vaccines in different ways [14], which has permitted ongoing assessment of the best means of using these vaccines, and their use continues to be refined [19].

In addition to the most efficient and effective use of the vaccine from an immunological point of view, health authorities have to consider many factors when planning population-scale interventions, including practical, economic, and political constraints [20]. When MCC vaccines were first introduced, these decisions had to be made in the absence of population-level information and inevitably involved an element of compromise. In the United Kingdom, for example, a priority was to integrate the new vaccine into the infant immunization schedule of 2, 3, and 4 months, but the occurrence of high-profile disease outbreaks in schools and universities necessitated a “catch-up” campaign of a single dose for all persons aged ≤18 years, which was administered through schools [11, 21]. This campaign was later extended to 24 years of age, but because this could not be implemented through schools, coverage was much lower. The catch-up campaign proved essential, because it subsequently became apparent that those immunized at 2, 3, and 4 months of age were not protected beyond 1 year of life [22], and substantial “vaccine failures” were probably only prevented by the herd immunity (also referred to as “community immunity” and “herd protection”) generated by immunizing teenagers, among whom most asymptomatic transmission occurred [23].

The Netherlands, which implemented the vaccine later and with more precise information, some of it from the United Kingdom experience, adopted a very different schedule, immunizing those aged >14 months with a single dose, employing a community approach and relying on herd immunity to protect the younger individuals [24]. In Canada, a range of different immunization protocols were adopted. Combined with the United Kingdom experience, the Dutch and Canadian surveillance data confirm that vaccine schedules that include both a dose after 1 year of age (at 12–14 months) and an adolescent booster are highly effective.

Fifteen years after the first introduction of MCC vaccines, schedules are still being modified in light of the insights obtained from the analysis of high-quality disease surveillance, immunological studies, and, importantly, surveys of carriage prevalence [25]. One conclusion is that these are close to being ideal vaccines; in addition to being very well tolerated, they elicit such strong responses that several different schedules are capable of achieving substantial reductions in disease that can last for at least 10 years. The Netherlands schedule has stood the test of time, although a teenage booster dose may be required for those routinely immunized at 14 months of age, whereas the United Kingdom schedule has undergone several changes, moving away from the approach of immunizing at 2, 3, and 4 months to immunizations later in life, with an increase emphasis on protection from herd immunity [26]. As reported in this journal [27] and elsewhere [28] last year, experience with MCC vaccines were also highly influential in designing and implementing the meningococcal serogroup A (MenAfriVac) vaccine in the meningitis belt of Africa, where the approach taken was to immunize everyone aged 1–29 years [29]. In all these cases, however, ongoing surveillance remains important to ensure that the reductions in disease incidence are maintained.

With the current interest in the implementation of vaccines against serogroup B meningococcus [30–32], it is perhaps worth closing with a reflection on what the experience with MCC vaccines does and does not tell us about meningococcal vaccination in general. It is clear that the high levels of immunity generated against the capsular polysaccharide affect carriage, and if this is achieved in those age groups where transmission is highest [33], significant herd immunity can be generated to protect the unvaccinated. This has also been observed with the *H. influenzae* type b vaccine and conjugate pneumococcal polysaccharide vaccines [9]; however, it is by no means inevitable that all vaccines designed against the meningococcus will have this effect. In particular, protein-based vaccines have not been shown to affect carriage so strongly or for so long, and the antigens they target are appreciably more variable than the meningococcal capsular polysaccharides. In the absence of conjugate polysaccharide vaccines against all meningococcal serogroups, meningococcal disease is unlikely to be completely eradicated, although reductions in disease levels are, perhaps, achievable by the judicious use of noncomprehensive vaccines [34].
